# Identification and phylogenetic characterization of novel hunnivirus recombinant strains in cattle from Guangxi, China

**DOI:** 10.3389/fcimb.2025.1559722

**Published:** 2025-03-06

**Authors:** Guangxin Zhang, Yuhang Luo, Jiajie Li, Chang Cui, Kang Ouyang, Ying Chen, Zuzhang Wei, Yifeng Qin, Qingting Dong, Yan Pan, Weijian Huang

**Affiliations:** ^1^ Laboratory of Animal Infectious Diseases and Molecular Immunology, College of Animal Science and Technology, Guangxi University, Nanning, China; ^2^ Guangxi Vocational University of Agriculture, Nanning, China; ^3^ Guangxi Key Laboratory of Animal Reproduction, Breeding and Disease Control, Nanning, China; ^4^ Guangxi Zhuang Autonomous Region Engineering Research Center of Veterinary Biologics, Nanning, China

**Keywords:** hunnivirus, cattle, recombinant, phylogenetic characterization, diarrhea

## Abstract

**Introduction:**

Hunnivirus (HuV), a member of the Picornaviridae family, is a single-stranded RNA virus associated with gastrointestinal issues in animals and poses potential zoonotic risks. While HuV has been detected in various animals, its prevalence and genetic characteristics in cattle remain poorly understood.

**Methods:**

From 2021 to 2023, we collected 1,017 fecal samples from cattle across Guangxi, China, and analyzed them for HuV using RT-PCR. Phylogenetic and sequence analyses were conducted to assess the virus’s genetic diversity and potential recombination events. Additionally, five HuV-positive samples were selected for whole-genome amplification and sequencing.

**Results:**

The overall prevalence of HuV was 3.05%, with significantly higher detection rates in diarrheic cattle (9.59%) compared to healthy cattle (2.54%). Regional prevalence varied, with the highest in Liuzhou (5.66%) and the lowest in Nanning (1.51%). Phylogenetic analysis identified a novel recombinant strain with distinct evolutionary patterns in the P3 genomic region. Sequence analysis revealed low homology in the VP1 and P1 regions compared to known genotypes, suggesting the classification of these strains as a new genotype. Additionally, the 5’ untranslated region (5’UTR) analysis confirmed the presence of type II Internal Ribosome Entry Sites (IRES), showing up to 91.8% nucleotide similarity with human parechovirus HPeV-3.

**Discussion:**

These findings reveal significant genetic diversity and regional adaptation of HuV in cattle populations. The virus is associated with gastrointestinal symptoms, especially in areas with suboptimal farming conditions, and exhibits a potential for zoonotic transmission. This study provides a foundation for further research into the virus’s pathogenicity and zoonotic risk, highlighting the need for continued surveillance to monitor its spread and evolution.

## 1 Introduction

Hunnivirus(HuV) is a single-stranded positive-sense RNA virus belonging to the genus HuV A within the Picornaviridae family, characterized by high genetic variability (Du et al., 2016; Walker et al., 2022). Its genome typically comprises a 5’ untranslated region (UTR), an open reading frame (ORF) that encodes structural and non-structural proteins, and a 3’ UTR (Firth et al., 2014). HuV occupies a unique taxonomic position within the Picornaviridae family. The structural proteins encoded by its genome include VP1, VP2, VP3, and VP4, while the non-structural proteins include 2A, 2B, 2C, 3A, 3B, 3C, and 3D (Liu et al., 2022; Zhu et al., 2024). Phylogenetic analyses of HuV have demonstrated its significant cross-species transmission capability, reflecting notable genetic diversity across different hosts (Lu et al., 2019).

The prevalence of HuV in various hosts displays significant regional variations both domestically and internationally. Initially identified in sheep in Northern Ireland in 1965, HuV has since been detected in cattle and sheep in Hungary (Isidan et al., 2019; Reuter et al., 2012), rodents in the USA and China (Du et al., 2016; Firth et al., 2014), pangolins in Vietnam (Lu et al., 2021), diarrheic cattle in Turkey, and cats in Guangdong, China. Epidemiological surveys in Hungary have shown detection rates of 2.1% in cattle and 1.8% in sheep. In New York City, USA, the detection rate in rodents was 6.3%, suggesting that these animals may serve as important reservoir hosts for the virus. Domestically, since the first isolation from water buffalo in Guangxi in 2021, studies have indicated a prevalence rate of 3.05% in the cattle population of the region, with a higher detection rate in diarrheic cattle (9.59%) (Liu et al., 2022). Furthermore, the detection rate in healthy cattle stands at 2.54%. Across seven sampling areas in Guangxi, the highest positivity rate was 5.66%, while the lowest was 1.89%, indicating geographical variations in the virus’s distribution. HuV demonstrates significant cross-species transmission capabilities, particularly among livestock and wildlife, which enhances its potential threat as a zoonotic disease (Shi et al., 2022).

The cross-species transmission capability of HuV may be associated with its highly variable genome and the virus’s ability to adapt to different hosts. Its widespread dissemination among rodents, livestock, and wildlife indicates that HuV can adapt to a variety of ecological niches. This adaptability may also be linked to the role of its non-structural proteins in evading host immune responses, particularly the functions of the 2C and 3D proteins in inhibiting type I interferon responses. Additionally, evolutionary analyses of HuV reveal frequent genetic recombination, which may be one of the key factors enabling successful cross-species transmission and ongoing evolution of the virus (Liu et al., 2024; Yang et al., 2022).

Despite these implications, research on HuV pathogenicity remains limited. Current studies have focused primarily on veterinary impacts, with scant data on its interaction with human cells or immune evasion strategies. Such findings highlight the urgency of investigating HuV’s zoonotic potential, particularly in regions like Guangxi, where close human-animal interactions and mixed farming systems may facilitate cross-species transmission (Tian et al., 2022; Wang et al., 2024).

This study aims to evaluate the epidemiological characteristics of HuV in Guangxi’s cattle population, utilizing phylogenetic analysis to explore its genomic features and evolutionary dynamics. By analyzing 1,017 cattle fecal samples with MEGA 7.0 software, the study identifies the virus’s prevalence rates and assesses potential risk factors affecting its transmission, such as environmental conditions, host immunity, and co-infections. These findings aim to enhance understanding of HuV’s cross-species transmission potential and support the development of effective control measures.

## 2 Materials and methods

### 2.1 Sample collection

From 2021 to 2023, this study was conducted across seven representative regions in Guangxi, including Nanning, Liuzhou, Guilin, Baise, Wuzhou, Yulin, and Hechi, to ensure a geographically representative sample distribution for assessing the prevalence and regional variations of HuV. A random sampling strategy was employed to minimize selection bias and ensure the accuracy and reliability of the results. A total of 1,017 cattle fecal samples, including 73 from diarrheic cattle, were collected following standardized procedures. Samples were immediately sealed in sterile containers, transported on ice to the laboratory within a few hours, and stored at -80°C until further processing. For sample preparation, fecal specimens were diluted in Dulbecco’s phosphate-buffered saline (DPBS) containing antibiotics and antifungal agents. The diluted samples underwent three freeze-thaw cycles, followed by centrifugation at 10,000 × g for 10 minutes at 4°C. The resulting supernatants (1 mL aliquots) were collected and stored at -40°C for subsequent RNA extraction and analysis. The sampled cattle populations included cover consecutive, ranging from large-scale farms to small family-owned operations, to comprehensively reflect the prevalence of HuV across the region (Luo et al., 2024).

### 2.2 RNA extraction and RT-PCR detection

In this study, RNA extraction was performed using the standardized Trizol method to efficiently extract viral RNA from fecal samples. The RNA extraction process was carried out strictly according to the operational protocols to ensure the accuracy and reliability of downstream experiments. The extracted RNA was dissolved in RNase-free DEPC water and immediately stored at -80°C to prevent degradation. For RT-PCR detection, HuV-specific primers targeting the 3CD gene region were used to ensure high specificity and sensitivity for the target virus. The RT-PCR reaction mix included 5× M-MLV Buffer, dNTPs, RNase inhibitor, M-MLV reverse transcriptase, random primers, and template RNA. Reverse transcription was performed at 42°C for 1 hour to generate cDNA. The resulting cDNA was directly used for subsequent PCR amplification (**Supplementary Table 1**). PCR amplification conditions consisted of an initial denaturation at 94°C for 5 minutes, followed by 35 cycles of denaturation at 94°C for 30 seconds, annealing at 55°C for 30 seconds, and extension at 72°C for 45 seconds, with a final extension at 72°C for 10 minutes. Amplification products were analyzed by electrophoresis on a 1.5% agarose gel to verify the effectiveness of the RT-PCR detection and the presence of positive samples (Luo et al., 2023).

### 2.3 Whole genome amplification and sequencing

For a comprehensive genomic analysis of HuV, five RT-PCR-positive samples were randomly selected for whole-genome amplification. The viral genome was amplified in segments using specifically designed primers that ensured complete coverage and efficient amplification. Each segment was independently sequenced three times to enhance sequence accuracy. Following amplification, all PCR products were purified using a specialized purification kit to eliminate impurities, ensuring high-quality sequencing results. The purified products were then subjected to Sanger sequencing to obtain the complete viral genome sequences. The sequencing data were carefully quality-controlled and manually assembled was performed to guarantee data accuracy and completeness. ORFs were predicted using GeneMarkS (v4.28) with virus-specific parameters and validated via BLASTp (v2.12.0+) against NCBI’s RefSeq viral database. The 5’ and 3’ untranslated regions (UTRs) were identified through sequence alignment with the reference strain OK642419 using MAFFT (v7.505) and RNA structure prediction via RNAfold (v2.4.18), while terminal conservation was assessed by comparing 50-bp flanking regions across 12 related strains using Clustal Omega (v1.2.4). Genome annotation incorporated data from NCBI’s Conserved Domain Database (CDD), Pfam (v35.0), and InterProScan (v5.56-89.0), with non-coding RNA elements predicted using Rfam (v14.9). Functional characterization of structural proteins was conducted via TMHMM (v2.0) for transmembrane domains and SignalP (v6.0) for secretory signals.Secondary Structure Prediction and Comparative Analysis of HuV 5’UTR.

To determine the genetic characteristics of HuV, evolutionary and phylogenetic analyses were conducted using SeqMan and MegAlign (DNASTAR, Madison, USA) to align and compare genome sequences and deduced amino acid sequences. Multiple sequence alignments were performed between the identified strains and reference sequences retrieved from the GenBank database.For phylogenetic analysis, the most suitable evolutionary model was determined to optimize tree construction. Maximum likelihood (ML) phylogenetic trees were generated using the Tamura-Nei and Kimura 2-parameter models, implemented in MEGA X (http://www.megasoftware.net/) with 1,000 bootstrap replicates. Recombination events in the HuV genome were identified using the Recombination Detection Program (RDP5.3). Seven distinct algorithms—RDP, GENECONV, MaxChi, Bootscan, SiScan, Chimaera, and 3Seq—were applied with default parameters, and only recombination events supported by at least six methods (P < 10⁻^6^) were considered significant. Identified recombination events were further validated using SimPlot v3.5.1 (Johns Hopkins University, Baltimore, MD, USA) under default settings.

To elucidate the structural characteristics of the 5’ untranslated region (5’UTR) of HuV and to compare these features with those of other picornaviruses (PVs), we conducted a detailed analysis of the 5’UTR sequences from five HuV strains isolated in our study. The secondary structures of the 5’UTRs were predicted using the Mfold web server, which utilizes thermodynamic algorithms to model likely RNA folding patterns at physiological temperatures. This approach allowed us to assess potential structural motifs that could influence viral virulence, tropism, and replication efficiency by facilitating comparisons with known 5’UTR structures in the PV family.

### 2.4 Co-infection with other pathogens

To investigate the association between HuV and viral diarrhea in cattle, as well as co-infection with other diarrhea-associated viruses, we performed RT-PCR on HuV-positive samples to detect six common bovine enteric viruses: bovine enterovirus (Luo et al., 2023), bovine coronavirus (Kia et al., 2021), bovine astrovirus (Fang et al., 2021; Kia et al., 2021), bovine rotavirus (Ates and Yesilbag, 2023), bovine viral diarrhea virus (Jiang et al., 2024), and boosepivirus (Ji et al., 2023). Specific primers targeting conserved regions of these viruses were used to determine the presence and frequency of co-infections.

## 3 Results

### 3.1 Prevalence of HuV

**Figure 1:**
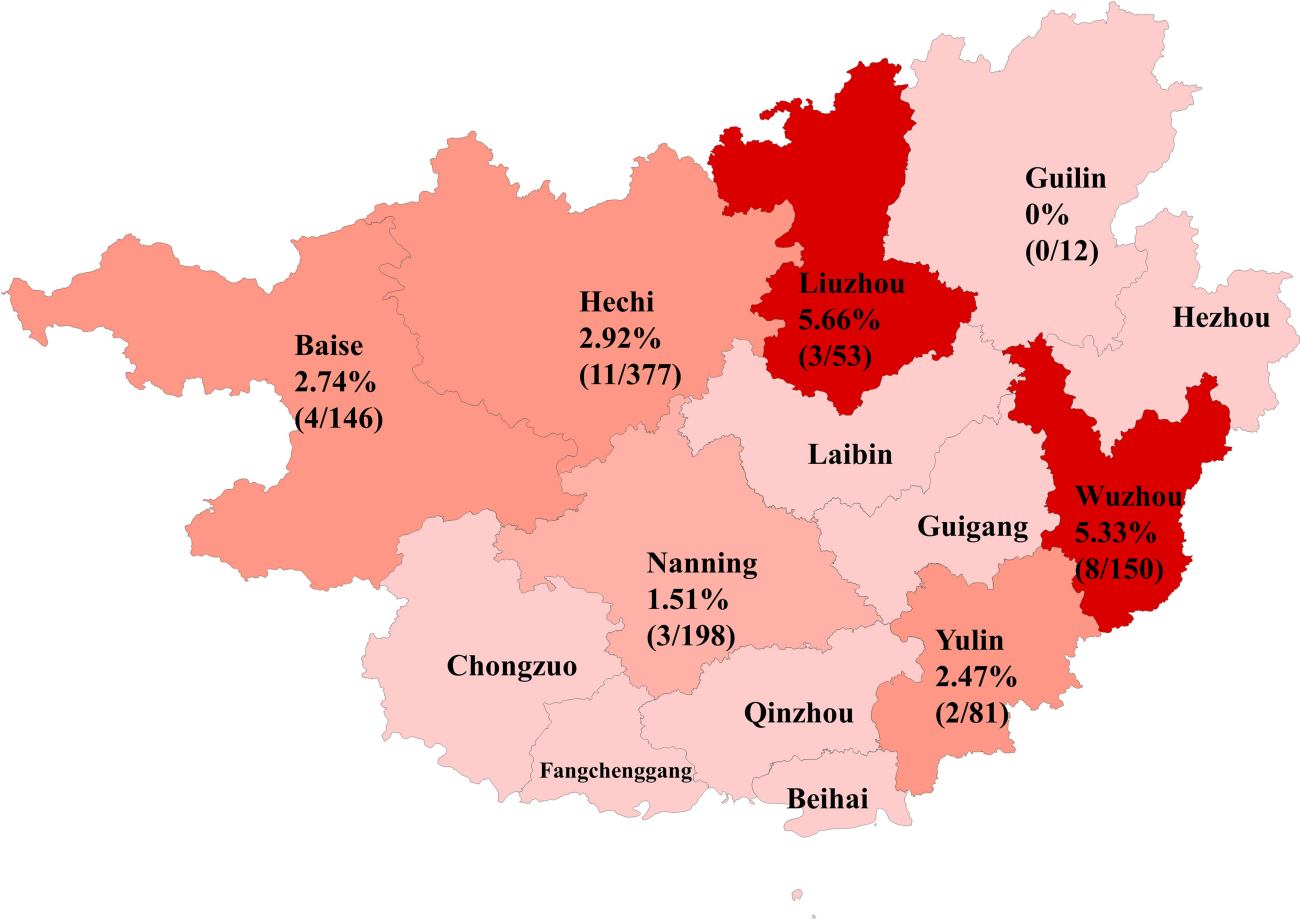
Positivity rates and geographic distribution of HuV in Guangxi Province.

This study examined 1,017 bovine fecal samples and found an overall HuV positivity rate of 3.05%. The positivity rate was significantly higher in diarrheic cattle at 9.59%, compared to 2.54% in healthy cattle, suggesting a potential association between HuV and the occurrence of diarrhea symptoms. There was also a noticeable geographic variability in HuV prevalence across different sampling areas, ranging from a high of 5.66% in Liuzhou to a low of 1.51% in Nanning. These differences likely reflect variations in breeding conditions, environmental factors, and the health status of the cattle populations in each area. The detailed positivity rates by area were as follows: Liuzhou 5.66%, Wuzhou 5.33%, Hechi 2.92%, Baise 2.74%, Yulin 2.47%, Nanning 1.51% and Guilin 0% (**Table 1**, **Figure 1**).

**Table 1:** Statistics of sample types and positive detection rate in various regions of Guangxi.

Location	Number of samples		Total	Number of positive samples		Total positives samples	Positive rate (%)		Total positive rate (%)
	Healthy	Diarrhoea		Healthy	Diarrhoea		Healthy	Diarrhoea	
Nanning	190	8	198	2	1	3	1.05	12.5	1.51
Wuzhou	139	11	150	7	1	8	5.03	9.1	5.33
Hechi	341	36	377	8	3	11	2.35	8.3	2.92
Baise	138	8	146	3	1	4	2.17	12.5	2.74
Guilin	12	0	12	0	0	0	0	0	0
Yulin	77	4	81	2	0	2	2.6	0	2.47
Liuzhou	47	6	53	2	1	3	4.26	16.6	5.66
Total	944	73	1017	24	7	31	2.54	9.59	3.05

### 3.2 Genome amplification and sequencing results

**Figure 2:**
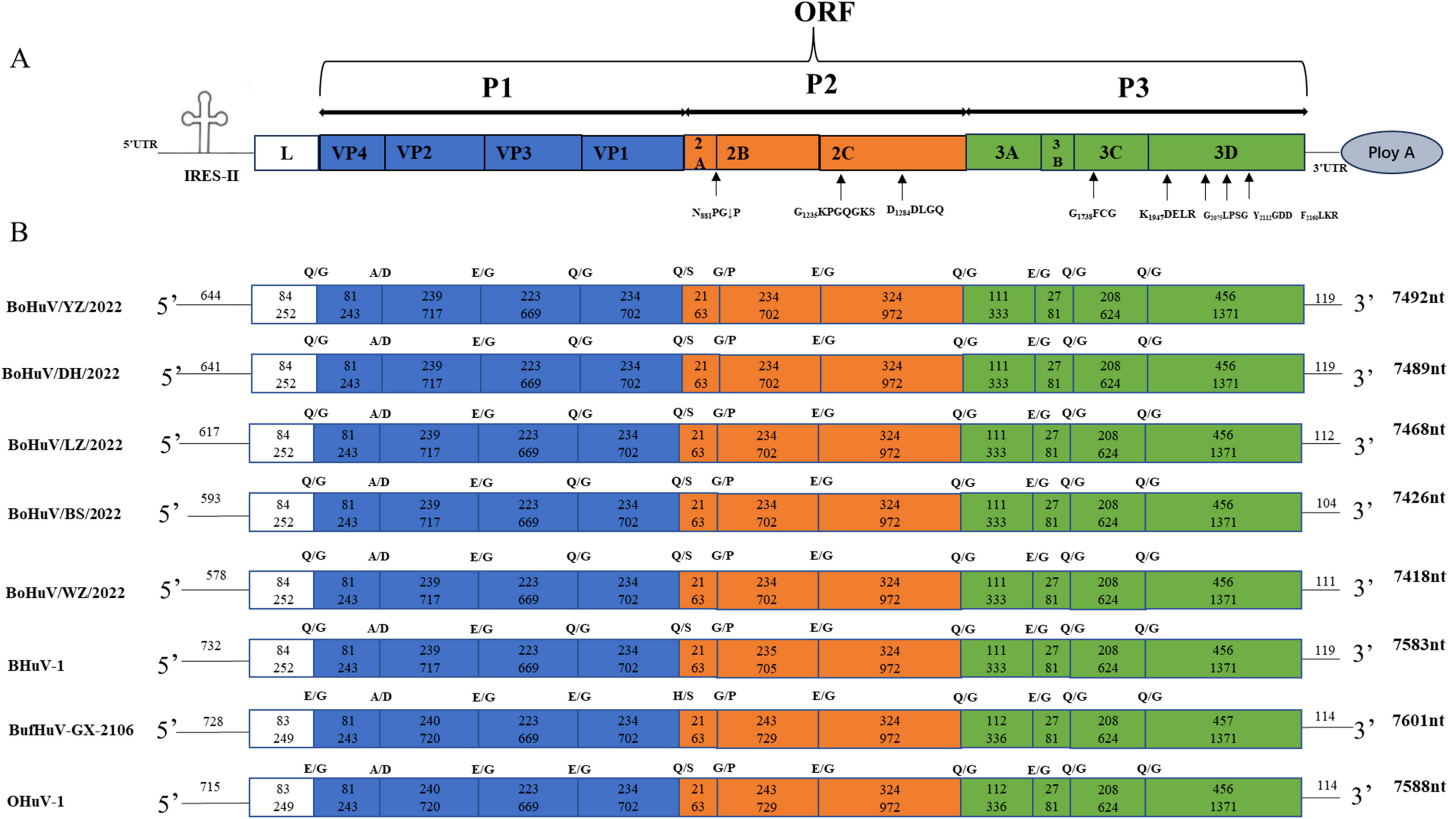
Schematic diagram of the complete genome structure of HuV. The full genome structure consists of a large ORF, 5’ UTR, 3’UTR, and PolyA tail. P1 represents the viral structural proteins, while P2 and P3 represent the non-structural proteins. The cloverleaf structure indicates the IRES element in the 5’ UTR. Predicted active sites within the large ORF coding region are indicated near the arrows. From top to bottom, the genomes represent the five HuV strains identified in this study, the BufHuV-GX-2106 strain previously isolated in our laboratory, and two reference strains from the GenBank database: bovine BHuV (accession no. JQ941880) and ovine OHuV (accession no. HM153767). The numbers within circles indicate the length and number of amino acids for each gene. Predicted cleavage sites between the referenced HuV genotypes are indicated above the coding regions.

The complete genomes were successfully amplified and sequenced from five HuV-positive samples. The genome lengths ranged from 7400 to 7600 nucleotides and featured typical ORFs and untranslated regions (5’ and 3’ UTRs). The genomic structure included a 6729 nt ORF encoding structural proteins P1 (VP4, VP2, VP3, VP1) and non-structural proteins P2 (2A, 2B, 2C) and P3 (3A, 3B, 3C). Specific genome lengths of the strains were 7492 nt, 7489 nt, 7468 nt, 7426 nt, and 7418 nt, respectively. The 5’ UTR lengths ranged from 579 nt to 645 nt, and the 3’ UTR lengths from 104 nt to 119 nt across different strains. Genomic analysis revealed conserved protease cleavage sites across all strains. Predicted cleavage sites included A/D for VP4/VP2, E/G for VP2/VP3, 2B/2C, and 3A/3B, Q/G for L/VP4, VP3/VP1, 2C/3A, 3B/3C, 3C/3D, Q/S for VP1/2A, and G/P for 2A/2B. Notably, the L protein lacked the GXCG motif (where x represents a non-conservative amino acid), and the P2 region encoded a 2A non-structural protein containing an N_881_PG↓P motif (↓ represents the ribosomal slippage site). The GXXGXGKS (G_1235_KPGQGKS) motif in 2C suggested NTP binding, and the DDLXQ (D_1284_DLGQ) motif indicated helicase activity. In the P3 region, the 3Cpro protein’s active site cysteine was part of the GXCG (G_1738_FCG) motif, and the 3Dpol contained a Y_2112_GDD motif. Additionally, conserved active sites in 3Dpol included K_1947_DELR, G_2075_LPSG, and F_2160_LKR (**Figure 2**).

### 3.3 HuV whole genome homology analysis

**Figure 3:**
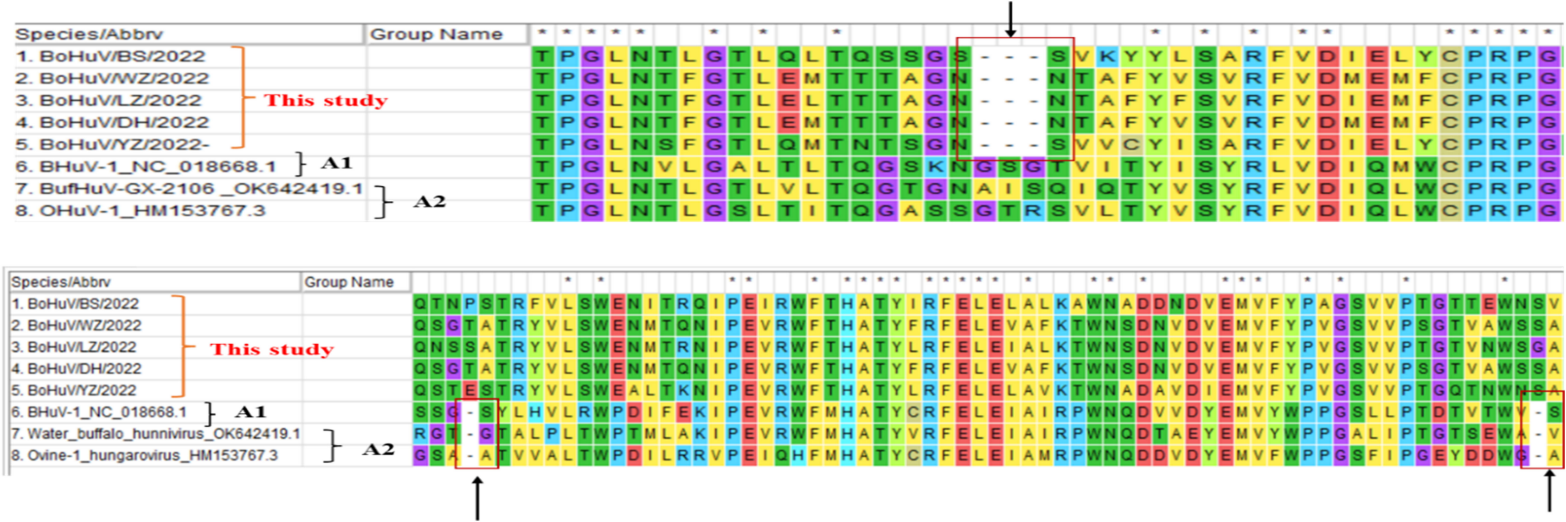
Comparative analysis of amino acid deletions in the VP1 region of HuV strains.

Homology comparisons were conducted for five HuV strains, assessing both nucleotide and amino acid sequences across the complete genome, and specific regions including L, P1, P2, P3, and VP1 (**Tables 2**, **3**). Results revealed nucleotide homology ranging from 72.6% to 95.8% across the complete genomes, with L protein varying between 75.3% and 98.4%, VP1 protein between 65.5% and 98.5%, P1 between 73.7% and 98.2%, P2 between 86.0% and 98.6%, and P3 non-structural protein between 90.9% and 93.1%. Amino acid homology across the entire genomes ranged from 71.9% to 91.7%. The highest nucleotide homology was observed between the strains BoHuV/YZ/2022 from Hechi and BoHuV/WZ/2022 from Wuzhou, reaching 95.8%, with amino acids at 91.7%. The lowest homology was between BoHuV/DH/2022 and BoHuV/BS/2022 from Hechi, with nucleotide and amino acid homologies at 83.9% and 71.9%, respectively (**Table 2**). Comparison with two representative HuV genomes in the NCBI database revealed nucleotide homology of 80.6%-81.3% with the Hungarian cattle HuVA1 type BHuV-1 (Accession No: JQ941880), and amino acids between 66.2%-68.2%. VP1 nucleotide sequences exhibited significant variability with low homology of 56.8%-58.3% and amino acids at 56.1%-57.8%. The P1 non-structural protein region nucleotides showed homology of 69.3%-70.9%, with amino acids at 65.2%-66.3%. Structural protein regions of P2 had nucleotide homologies of 84.9%-86.1% and amino acids of 78.4%-80.0%. The P3 structural protein showed high nucleotide and amino acid homology, ranging from 89.5%-91.1% and 79.9%-83.7%, respectively (**Table 3**). The VP1 protein of the five HuV strains exhibited unique features, including two additional amino acids at positions 63 and 128 and three deletions between positions 205 and 209 compared to BHuV-1, BufHuV-GX-2106, and OHuV-1 (**Figure 3**).

**Table 2:** Homology analysis of 5 strains of HuV genome sequences.

Nucleotide (nt %) and amino acid (aa %) homology											
		BoHuV-BS-2022		BoHuV-DH-202		BoHuV-LZ-2022		BoHuV-WZ-2022		BoHuV-YZ-2022	
		nt (%)	aa (%)	nt (%)	aa (%)	nt (%)	aa (%)	nt (%)	aa (%)	nt (%)	aa (%)
BoHuV-YZ-2022	Genome L VP1 P1 P2 P3	86.7 77.3 67.8 72.3 94.9 94.2	76.7 67.9 69.9 81.2 95.2 88.2	85.2 96.4 68.5 74.5 86.2 93.2	74.7 94.0 73.0 83.1 81.0 88.0	86.6 79.4 75.8 77.7 87.3 94.8	76.1 67.9 85.2 81.8 81.4 90.1	96.5 98.4 98.5 98.2 98.6 93.1	91.7 96.4 99.6 99.5 97.8 83.0	N-A N-A N-A N-A N-A N-A	N-A N-A N-A N-A N-A N-A
BoHuV-WZ-2022	Genome L VP1 P1 P2 P3	85.4 77.7 67.1 72.2 95.1 92.3	75.3 67.9 69.2 80.9 95.3 83.0	84.3 97.2 67.9 74.3 86.3 90.9	72.3 95.2 72.6 83.0 81.0 82.0	86.1 75.3 75.5 77.4 87.2 91.7	72.7 67.9 84.8 87.8 81.0 82.0	N-A N-A N-A N-A N-A N-A	N-A N-A N-A N-A N-A N-A	- - - - - -	- - - - - -
BoHuV-LZ-2022	Genome L VP1 P1 P2 P3	85.2 90.9 65.5 72.2 86.5 93.6	72.4 85.7 69.6 80.2 80.4 87.5	84.6 76.9 69.9 74.4 88.5 92.6	74.2 71.4 73.8 82.8 85.4 86.7	N-A N-A N-A N-A N-A N-A	N-A N-A N-A N-A N-A N-A	- - - - - -	- - - - - -	- - - - - -	- - - - - -
BoHuV-DH-2022	Genome L VP1 P1 P2 P3	84.1 79.3 68.4 73.2 86.0 92.8	71.9 71.4 74.7 80.9 80.4 87.3	N-A N-A N-A N-A N-A N-A	N-A N-A N-A N-A N-A N-A	- - - - - -	- - - - - -	- - - - - -	- - - - - -	- - - - - -	- - - - - -

N-A indicates data not comparable; “ - “ indicates that the data are presented repeatedly.

**Table 3:** Homology analysis of 5 HuV genomes and representative strains of different genotypes of NBCI.

		Nucleotide (nt %) and amino acid (aa %) homolog																	
		BHuV-1 (HuV A1)						BufHuV-GX-2106(HuV A2)						OHuV-1(HuV A2)					
		Genome	L	VP1	P1	P2	P3	Genome	L	VP1	P1	P2	P3	Genome	L	VP1	P1	P2	P3
BoHuV/YZ/2022	nt aa	81.0 67.7	88.1 79.8	56.8 57.8	70.2 65.2	85.6 79.4	90.7 82.3	72.9 54.0	64.8 53.6	59.2 56.5	68.0 64.4	73.7 64.5	79.4 61.4	73.0 54.1	66.4 56.0	57.2 56.1	68.8 63.7	74.3 65.7	79.6 61.7
BoHuV/WZ/2022	nt aa	80.6 66.2	88.9 81.0	57.0 57.4	69.9 65.3	85.3 78.4	89.5 79.9	73.0 54.2	64.8 53.6	58.8 56.1	67.7 64.3	73.7 64.0	80.0 62.2	73.0 53.8	66.4 56.0	57.2 55.7	68.5 63.7	74.5 65.7	79.5 60.8
BoHuV/LZ/2022	nt aa	81.0 68.0	70.5 60.7	57.0 57.8	69.3 65.2	86.1 79.7	91.1 83.0	73.1 55.1	61.4 48.8	59.1 56.5	68.0 64.7	73.1 63.8	80.7 62.9	73.0 55.2	62.5 47.6	56.1 56.1	67.5 63.9	73.2 64.5	80.5 62.9
BoHuV/DH/2022	nt aa	81.3 68.2	88.9 83.3	57.1 56.1	70.3 66.3	84.9 80.0	90.9 82.7	73.4 55.3	65.2 56.0	59.2 54.0	68.2 64.9	73.1 63.7	80.8 62.9	73.3 54.8	66.0 56.0	57.2 54.0	68.0 64.7	73.4 66.7	80.3 62.1
BoHuV/BS/2022	nt aa	81.0 67.0	74.9 63.1	58.3 57.0	70.9 65.8	85.5 78.9	91.0 83.7	73.3 54.7	63.3 48.8	59.9 56.5	68.6 64.9	73.5 63.5	80.5 62.7	73.6 55.1	63.7 46.4	58.8 56.5	69.1 65.2	74.2 66.0	80.5 63.0

**Figure 4:**
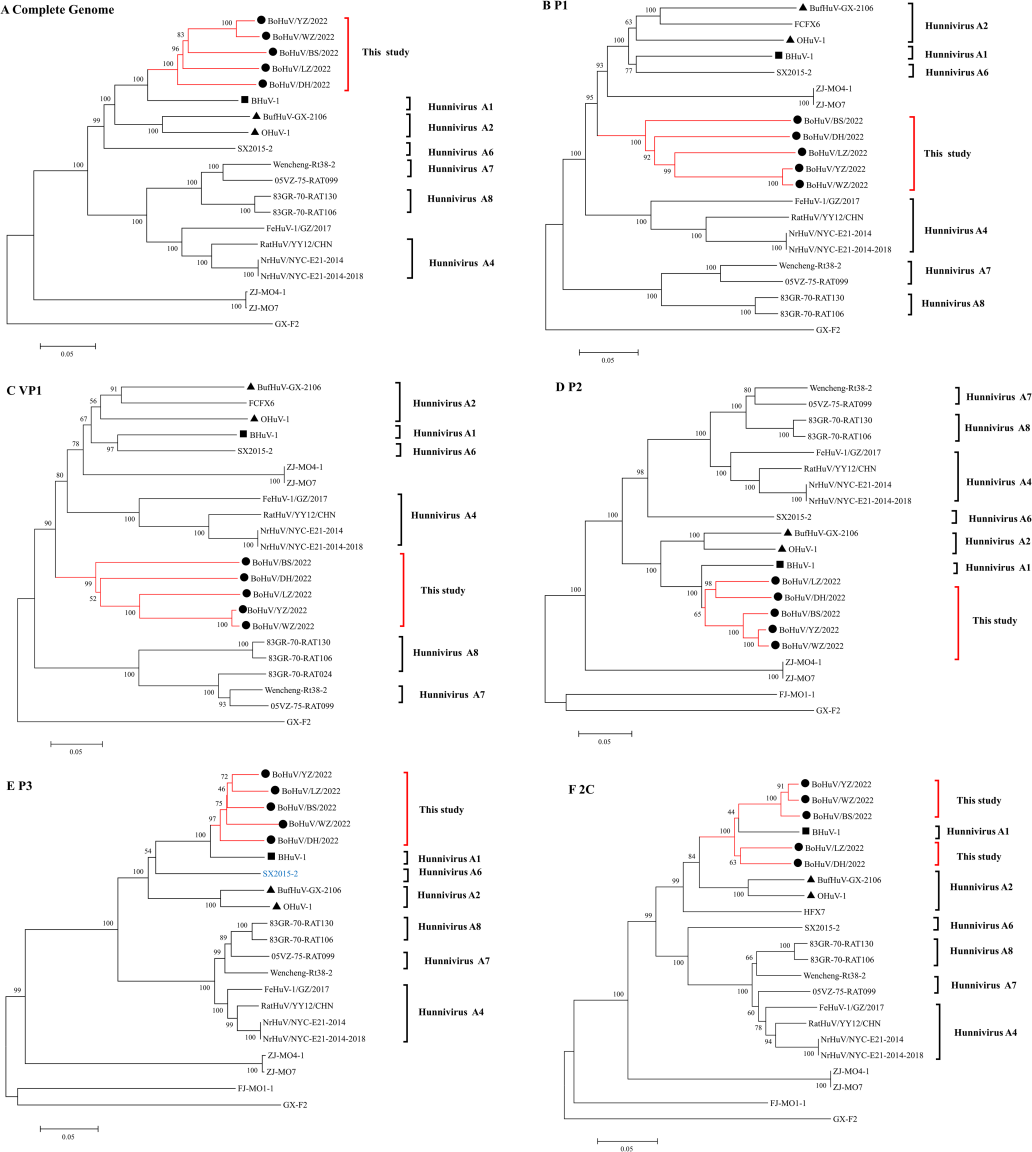
Phylogenetic trees of HuV strains based on different genomic regions. Phylogenetic analysis was conducted for HuV strains using nucleotide sequences from **(A)** the complete genome, **(B)** the P1 gene segment, **(C)** the VP1 gene segment, **(D)** the P2 gene segment, **(E)** the P3 gene segment, and **(F)** the 2C gene segment. Symbols indicate different HuV strains: “●” represents the five strains identified in this study, “▴” represents HuV A2 genotypes OHuV-1 and BufHuV-GX-2106, and “■” represents HuV A1 genotype BHuV-1.

Phylogenetic analysis of five HuV strains compared with 14 domestic and international reference strains and one laboratory strain revealed distinct evolutionary patterns. The study strains clustered closely with Hungarian cattle HuVA1 (BHuV-1), HuVA2 (OHuV-1), and Chinese water buffalo HuVA2 (BufHuV-GX-2106) in the full genome, P2, P3, and 2C regions, forming a larger branch, while in the full genome, P2, and P3 trees, they formed a distinct smaller cluster. In the 2C region, they clustered with BHuV-1, suggesting a shared evolutionary pattern. Additionally, the study strains clustered with SX2015-2, a strain from rodent feces in China (accession number KX156157), in the full genome and P3 trees. However, in the VP1 and P1 regions, the study strains formed a unique branch, showing distant relationships with BHuV-1, OHuV-1, BufHuV-GX-2106, and other genotypes, with amino acid homologies notably lower, ranging from 54-57.8% for VP1 and 63.7-66.3% for P1. BoHuV/WZ/2022 and BoHuV/YZ/2022 clustered closely in the full-length, P1, VP1, P2, and 2C regions, with amino acid homology of 91.7%, 99.6%, 99.5%, 97.8%, and 95.6%, respectively. In contrast, BoHuV/LZ/2022 and BoHuV/YZ/2022 showed closer clustering in the P3 region, with homologies of 90.1% and 83.0%, suggesting different evolutionary patterns. Despite geographical and temporal distances, the Guangxi strains were phylogenetically closer to BHuV-1 than to the Guangxi water buffalo strain BufHuV-GX-2106. The five strains formed a distinct cluster in the P1 and VP1 trees, highlighting their genetic divergence from other genotypes. The highly variable and immunodominant VP1 capsid protein further supports their classification as a new subtype within the HuV genus (**Figure 4**, **Table 3**).

### 3.4 Recombination analysis

**Figure 5:**
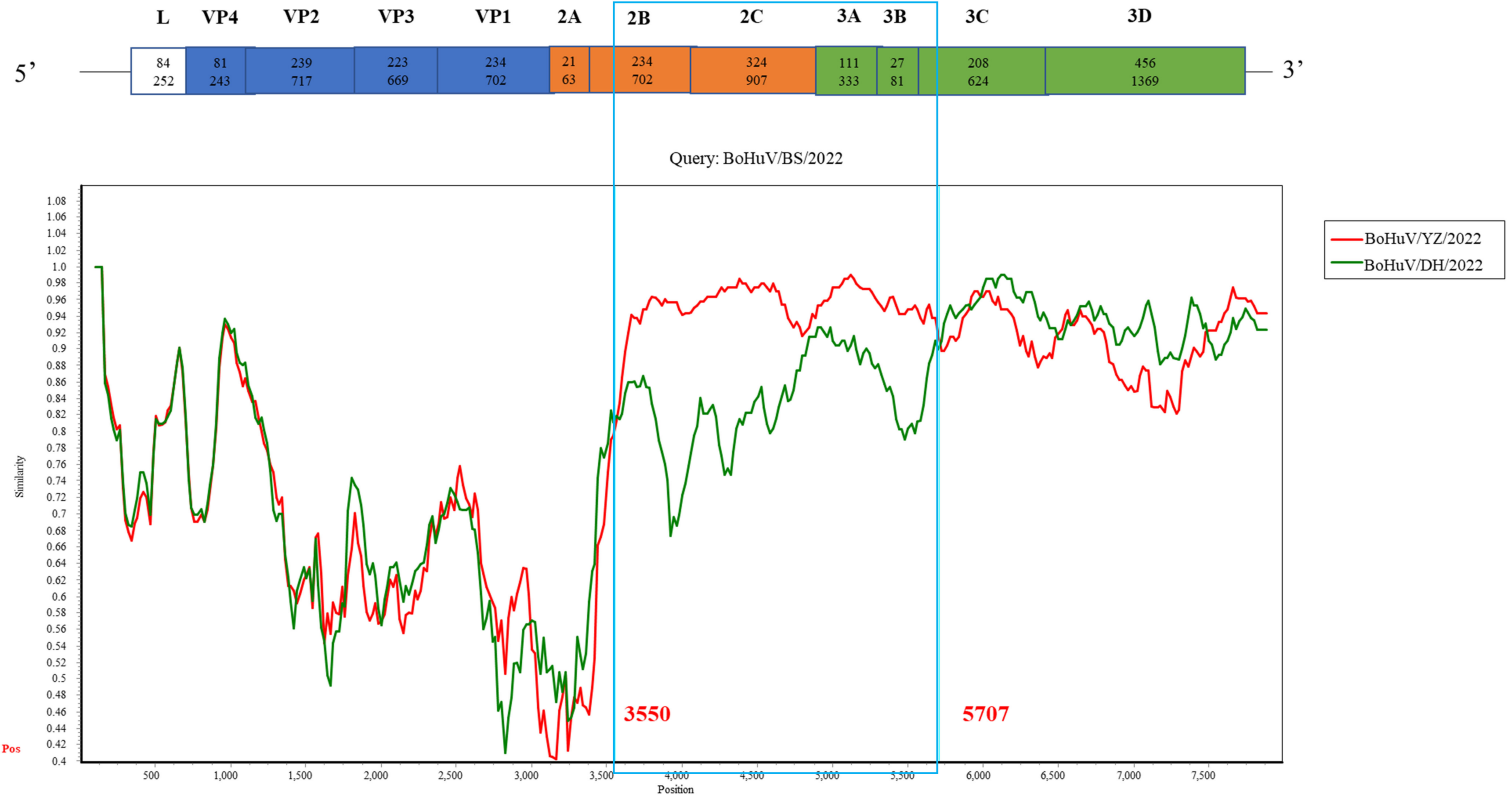
Simplot recombination analysis of BoHuV/BS/2022. Comparison of genomic similarity between BoHuV/BS/2022 and BoHuV/DH/2022 (green) and BoHuV/YZ/2022 (orange) Potential recombination breakpoints are represented by blue vertical lines and labeled with nucleotide positions.

Recombination analysis was performed on five HuV strains from this study, alongside 11 publicly available genome sequences and one previously isolated laboratory strain, using algorithms such as RDP, GENECONV, MaxChi, BootScan, Chimaera, SiScan, and 3Seq. No recombination events were detected between the study strains and other known strains. However, a potential recombination event was identified within BoHuV/BS/2022, scoring 0.537 with six algorithms indicating p-values less than 1×10^-6. Further analysis using Simplot suggested recombination breakpoints between bases 3550 and 5707 in the 2B-3C region. Phylogenetic analysis of this region showed the closest relationship between BoHuV/BS/2022 and BoHuV/YZ/2022, consistent with the recombination findings, suggesting BoHuV/BS/2022 likely resulted from recombination between BoHuV/YZ/2022 and BoHuV/WZ/2022 (**Figure 5**).

### 3.5 Structural analysis of HuV 5’UTR

**Figure 6:**
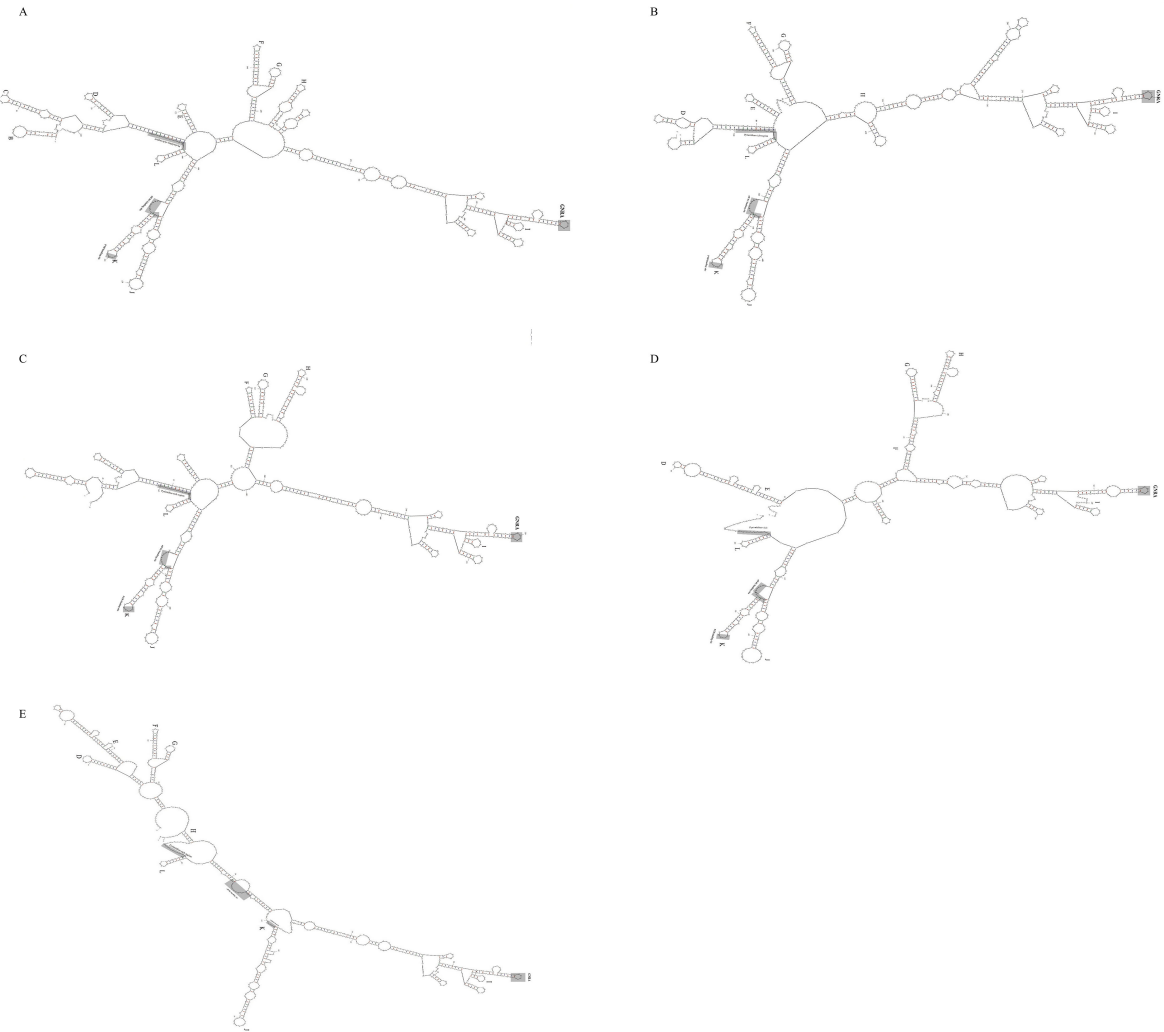
Predicted secondary structure of the 5’UTR ofBoHuV/YZ/2022 **(A)**, BoHuV/WZ/2022 **(B)**, BoHuV/LZ/2022 **(C)**, BoHuV/BS/2022 **(D)** and BoHuV/DH/2022 **(E)**.

The 5’ untranslated regions (5’UTR) of five HuV strains analyzed in this study show translation initiation from the eleventh AUG codon, with a preceding polypyrimidine tract (UUUUCCUUUU) located 17-19 nt upstream, fitting a Yn-Xm-AUG pattern. Mfold-predicted secondary structures indicate stable formations with minimum free energies ranging from -180.67 to -215 kcal/mol, suggesting the presence of type II Internal Ribosome Entry Sites (IRES) similar to those in EMCV and FMDV. These IRES elements contain five core structural domains (H to L) and motifs including GNRA and binding sites for transcription factors and PTB. The sequence analysis reveals high structural similarity between the core domains of these strains and those of EMCV, FMDV, BHuV-1, BufHuV-GX2106, and high nucleotide homology with human parechoviruses, highlighting conserved evolutionary features (**Figure 6**).

### 3.6 Co-infection rates of HuV with other diarrheal pathogens in cattle

This study assessed the prevalence of co-infections between HuV and other diarrheal pathogens in cattle. The analysis revealed that bovine enterovirus had the highest co-infection rate with HuV at 45.2%, followed by bovine astrovirus at 32.1%, bovine rotavirus at 18.7%, and bovine coronavirus at 12.5%. In contrast, no positive cases of co-infection with bovine viral diarrhea virus (BVDV) or boosepivirus were detected, suggesting potential differences in the epidemiological distribution or interaction dynamics of these pathogens with HuV.

## 4 Discussion

The study investigated the differential prevalence of HuV across various cattle herds in Guangxi, analyzing the influences of geographical factors, farming conditions, and the health status of cattle on its spread. The findings indicated that HuV infections are more prevalent in regions with less intensive farming practices and in cattle that are used both for labor and meat, which typically have lower immunity. For instance, native yellow cattle, often kept in small-scale, free-range conditions, exhibited higher infection rates compared to more commercially farmed Simmental and water buffalo, which benefit from systematic vaccination and better-managed farming practices. The geographical distribution of HuV in Guangxi showed significant heterogeneity, with detection rates ranging from 0% in Guilin to 5.66% in Liuzhou. This variation may reflect differences in farming practices, environmental conditions, and cattle population dynamics. Liuzhou, with small-scale, free-range farming, had the highest prevalence (5.66%), while Nanning, with more commercial farms and stricter biosecurity measures, had the lowest (1.51%). These findings support the idea that less intensive farming systems, with lower herd immunity and veterinary oversight, may facilitate viral transmission (Holland, 1990). Additionally, the higher prevalence in diarrheic cattle (9.59%) compared to healthy cattle (2.54%) suggests an etiological link between HuV and gastrointestinal disease, especially in regions with suboptimal management. Climate and ecology likely influence viral persistence, with Guangxi’s humid subtropical climate enhancing viral survival in contaminated environments (Lenaker et al., 2017; Morin et al., 2018). Conversely, urbanized farming systems in Nanning may reduce cross-species transmission. Phylogenetic analysis of Guangxi strains clustered with Hungarian and Chinese rodent-derived HuV, suggesting historical cross-regional transmission. However, the lack of detection in Guilin, despite similar climate, calls for further exploration of local factors like cattle breed susceptibility, vector presence, or undetected co-infections.

The study also revealed higher co-infection rates of HuV with other diarrheal pathogens, notably bovine enterovirus and bovine astrovirus, which could exacerbate gastrointestinal symptoms and pose a threat to cattle health (Zhu et al., 2022). This suggests a potential link between HuV infections and clinical signs of diarrhea, underscoring the need for continuous monitoring and improved management practices in cattle farming to mitigate the economic impacts of gastrointestinal diseases in the livestock industry (Luo et al., 2023).

HuV, a non-enveloped, single-stranded RNA virus of the Picornaviridae family, encodes a single ORF that includes structural and non-structural proteins. Notably, the VP1 protein is the most variable and immunodominant in the capsid, serving as a key marker for phylogenetic analysis. Initially identified in Hungary, HuV has since been classified into several genotypes. This study’s analysis of five HuV strains shows low amino acid and nucleotide homology in the VP1 and P1 regions compared to other strains, with unique amino acid deletions relative to established HuV strains like BHuV-1 and BufHuV-GX-2106. Phylogenetic analysis positions these strains in a distinct branch, suggesting they represent a new HuV genotype. These findings underscore the need for ongoing genetic surveillance to elucidate HuV diversity and its implications for animal health.

The analysis of five HuV strains originating from various districts in Guangxi reveals notable genetic diversity and suggests regional transmission contributing to evolutionary variance. Notably, BoHuV/WZ/2022 from Wuzhou and BoHuV/YZ/2022 from Hechi exhibit close phylogenetic relationships yet differ in their P3 region evolution patterns, indicating strain adaptation due to geographical spread. Besides these two strains, the remaining show lower homology in the VP1 region across different strains, highlighting significant genetic variability within HuV. Phylogenetic trees constructed for full genome, VP1, P1, P2, and P3 suggest that while these strains cluster together in a distinct sub-branch, they also align closely with the Hungarian cattle strain BHuV-1 and distantly with the BufHuV-GX-2106 from Chinese water buffalo. This similarity may reflect a common ancestral lineage, cross-border livestock trade, or the role of wildlife reservoirs in viral dissemination. Interestingly, despite geographical and temporal distances, HuV sequences from Chinese rodent feces (SX2015-2) displayed closer relatedness to Hungarian strains (BHuV-1 and OHuV-1) than to HuV from Norwegian rats in the USA. Rodents, which are known reservoirs of various picornaviruses, could have facilitated long-distance viral spread through natural migration or human-mediated activities. This study underscores HuV’s capacity for global distribution and cross-species transmission, highlighting the need for ongoing surveillance of HuV epidemiology, especially in countries with extensive livestock industries like China, where current epidemiological data on HuV are lacking (Jimenez-Clavero et al., 2005).

Our study identifies, for the first time, a recombination event in the HuV strain BoHuV/BS/2022. Recombination has been recognized as a pivotal mechanism in the evolution of picornaviruses, often leading to the emergence of new genotypes and alterations in host range. Despite the absence of prior reports on recombination within HuV, this finding aligns with observations in other viruses where interspecies recombination facilitates adaptation to new hosts, as suggested by in the case of feline norovirus strain M49 (Takano et al., 2015). The recombination detected in HuV could similarly be considered an evolutionary adaptation mechanism, potentially enhancing its ability to spread and establish in cattle populations. Continued research and surveillance are crucial to further understand the role of recombination in the transmission dynamics and host adaptation of HuV.

The 5’ untranslated region (5’UTR) of Hunnivirus (HuV) harbors a type II Internal Ribosome Entry Site (IRES), characterized by five conserved core structural domains (H to L) and nucleotide motifs, including GNRA tetraloops and A-bulges critical for ribosome recruitment (Koirala et al., 2019; Leppek et al., 2018). Our study revealed that the IRES of Guangxi HuV strains shares up to 91.8% nucleotide similarity with human parechovirus HPeV-3, a pathogen linked to neonatal sepsis and encephalitis (Olijve et al., 2018). This striking homology suggests evolutionary convergence or shared ancestry with human-infecting parechoviruses, potentially enabling HuV to exploit similar translation initiation mechanisms. Notably, the type II IRES in HuV mirrors structural features observed in Ljungan/Sebokele-like picornaviruses recently identified in birds of prey (Pankovics et al., 2017). For instance, the falcon-derived Ljungan-like virus (falcon/HA18_080/2014/HUN) also possesses a type II IRES with conserved domains H–L and a pyrimidine-rich tract upstream of the initiation codon, sharing 79% nucleotide similarity to rodent-borne Ljungan viruses and HuV. Both studies highlight the conservation of critical IRES motifs, such as the A-bulge in domain J, which mediates interactions with eukaryotic initiation factor eIF4G—a mechanism essential for cap-independent translation in picornaviruses. However, differences exist in IRES-host adaptation strategies. While the falcon virus IRES retains structural fidelity to rodent Ljungan viruses, HuV’s IRES exhibits unprecedented similarity to human HPeV-3, implying distinct evolutionary pressures. The high nucleotide homology between HuV and HPeV-3 in domain D—a region critical for ribosome binding—suggests that HuV may utilize analogous translation machinery in human cells. In contrast, the avian Ljungan-like virus, despite IRES conservation, shows no evidence of human tropism, possibly due to divergent host-specific adaptations in non-structural regions (e.g., 2A protein variations).

## 5 Conclusion

This study provides a comprehensive analysis of HuV prevalence, genetic diversity, and evolutionary dynamics in cattle populations across Guangxi, China. Among 1,017 fecal samples, an overall prevalence of 3.05% was observed, with significantly higher rates in diarrheic cattle (9.59%) compared to healthy cattle (2.54%). Phylogenetic analysis identified a novel recombinant strain, exhibiting distinct evolutionary patterns in the P3 genomic region, and significant genetic divergence in the VP1 and P1 regions compared to known genotypes. Homology analysis revealed low nucleotide and amino acid similarity in key regions but demonstrated clustering with Hungarian and Chinese rodent strains, indicating potential cross-species transmission and regional adaptations. Additionally, analysis of the 5’UTR identified type II IRES elements with up to 91.8% nucleotide similarity to human parechoviruses, highlighting evolutionary links and functional relevance to viral replication. These findings underscore HuV’s genetic diversity, its association with diarrheal symptoms, and its potential for zoonotic transmission. The study emphasizes the importance of expanded surveillance and genomic studies to better understand the epidemiology, pathogenicity, and public health risks associated with HuV.

## 6 Limitations and future directions of the study

This study is limited by the number of samples and the geographical scope of sampling, which may affect the representativeness and generalizability of the results. Additionally, the pathogenic mechanisms of HuV remain unclear, and its specific impact on cattle health as well as its potential risk as a zoonotic agent require further investigation. While this research provides preliminary epidemiological data on HuV, future studies should expand the number of samples and broaden the sampling scope, particularly across different geographical regions and farming environments, to enhance the breadth and reliability of the findings. Moreover, further exploration into the pathogenic mechanisms of HuV, its interactions with the host immune system, and its implications for public health are crucial for developing effective prevention and control strategies.

## Data Availability

The datasets presented in this study can be found in online repositories. The names of the repository/repositories and accession number(s) can be found below: https://www.ncbi.nlm.nih.gov/genbank/, OQ790149 https://www.ncbi.nlm.nih.gov/genbank/, OQ790150 https://www.ncbi.nlm.nih.gov/genbank/, OQ790151 https://www.ncbi.nlm.nih.gov/genbank/, OQ790152 https://www.ncbi.nlm.nih.gov/genbank/, OQ790153.
