# Supplementary material for: Identification and phylogenetic characterization of novel hunnivirus recombinant strains in cattle from Guangxi, China
Source: Front Cell Infect Microbiol. 2025 Mar 6;15:1559722. doi: 10.3389/fcimb.2025.1559722 (PMC11922853; doi:10.3389/fcimb.2025.1559722)
Supplement: Supplementary file 1 [file Table1.docx]

Supplementary Table 1 The primers used for detecting different viruses.

| Primer name | Primes sequences (5´-3´) | Length of PCR  product (bp) |
| --- | --- | --- |
| BoHuV-F | GACAAYGGCATYTGGAAGAAAG | 378 |
| BoHuV-R | TGCAGCGACAGTCGTTCATTCAC |  |
| BCOV-F | ATGTCTTTTACTCCTGGTAAGCAATC | 774 |
| BCOV-R | ATGTCTTTTACTCCTGGTAAGCAATC |  |
| BEV-F | CCGACTCCGCACCGATACGTCG | 236 |
| BEV-R | CTCTCAGAGCTACCACTGGGGT |  |
| BPV-F | GCGAAAACACGACTTTG | 554 |
| BPV-R | GAGCCGTGTCACCAGTGTTA |  |
| BAstV-F | CGKTAYGATGGKACKATICC | 422 |
| BAstV-R | GGYTTKACCCACATICCRAA |  |
| BVDV-F | ATGCCCTTAGTAGGACTAGCA | 292 |
| BVDV-R | CAACTCCATGTGCCATGTACAGCAG |  |

Supplementary Table 2 Amplification primers for the whole genome of five Hunnivirus strains

| Virus name | Primer | Primer sequence ( 5 ' -3 ' ) | Product size |
| --- | --- | --- | --- |
| BoHuV/WZ/2022 | WZ-1F  WZ-IR | CTTACCCTTCATTTGTGAACCC  GAATTTTGCCACTGCTGGTTGTAA | 913 bp |
|  | WZ-2F  WZ-2R | TTGGATGTTGAGTCGACTATG  GCTGTCACAATAGGAGTGGTT | 1714 bp |
|  | WZ-3F  WZ-3R | GGTGGGTATTTTACAATTTGGCA  GCCAATGATTCTCCAAATGGATTGT | 1435 bp |
|  | WZ-4F  WZ-4R | CCACTTTCAGTGAATTCAGGTGACA  TCACATCTAGCCATGCGGTC | 1534 bp |
|  | WZ-5F  WZ-5R | TGCACAAGATCCACATGATGATG  TGCAGCGACAGTCGTTCATTCAC | 1206 bp |
|  | WZ-6F  WZ-6R | TGCGGAATCTGCCAAGACCTG  TTCAAGGTCATCAAAGCTAGGAAC | 1557 bp |
|  | WZ-7F  WZ-7R | CGATTCAACTCACATTTGGGATGA  TTACTCTGGGGAAAATTAACCCT | 665 bp |

Supplementary Table 2 Amplification primers for the whole genome of five Hunnivirus strains（Continued）

| Virus name | Primer | Primer sequence ( 5 ' -3 ' ) | Product size |
| --- | --- | --- | --- |
| BoHuV/WZ/2022 | WZ-1F  WZ-IR | CTTACCCTTCATTTGTGAACCC  GAATTTTGCCACTGCTGGTTGTAA | 913 bp |
|  | WZ-2F  WZ-2R | TTGGATGTTGAGTCGACTATG  GCTGTCACAATAGGAGTGGTT | 1714 bp |
|  | WZ-3F  WZ-3R | GGTGGGTATTTTACAATTTGGCA  GCCAATGATTCTCCAAATGGATTGT | 1435 bp |
|  | WZ-4F  WZ-4R | CCACTTTCAGTGAATTCAGGTGACA  TCACATCTAGCCATGCGGTC | 1534 bp |
|  | WZ-5F  WZ-5R | TGCACAAGATCCACATGATGATG  TGCAGCGACAGTCGTTCATTCAC | 1206 bp |
|  | WZ-6F  WZ-6R | TGCGGAATCTGCCAAGACCTG  TTCAAGGTCATCAAAGCTAGGAAC | 1557 bp |
|  | WZ-7F  WZ-7R | CGATTCAACTCACATTTGGGATGA  TTACTCTGGGGAAAATTAACCCT | 665 bp |

Supplementary Table 2 Amplification primers for the whole genome of five Hunnivirus strains（Continued）

| Virus name | Primer | Primer sequence ( 5 ' -3 ' ) | Product size |
| --- | --- | --- | --- |
| BoHuV/LZ/2022 | LZ-1F  LZ-IR | GGGACCACCACTTTCAGTGAC  GCACCACCATAAGCAGTAGCAT | 1031 bp |
|  | LZ-2F  LZ-2R | TGGTCAGACGCCAGAAGA  TGCAGTTGTGGGTGACCCAGGT | 1596 bp |
|  | LZ-3F  LZ-3R | TATGTGGCGGCATACACDCCACC  ACATCAATCCACTGGGCAAAATC | 1105 bp |
|  | LZ-4F  LZ-4R | CTGGGATGTACATAGATCCG  ACAACTGGTTGCTGTTGATATCC | 1311 bp |
|  | LZ-5F  LZ-5R | TGGTTTGATGAGATGCGGAAA  GGCTTCAATGGATCTGGTCCTG | 975 bp |
|  | LZ-6F  LZ-6R | TGGTGTGACCTTGCAATYGCAC  TGCAGCGACAGTCGTTCATTCAC | 1318 bp |
|  | LZ-7F  LZ-7R | TGACGGGCCGCTAGCCATGATTA  TTCAAGRTCATCAAAGCRGGGAAC | 1155 bp |
|  | LZ-8F  LZ-8R | CAGATACTTGCATATGGAGACG  TTACTCTGGGGAAAATTAACCCT | 520 bp |

Supplementary Table 2 Amplification primers for the whole genome of five Hunnivirus strains（Continued）

| Virus name | Primer | Primer sequence ( 5 ' -3 ' ) | Product size |
| --- | --- | --- | --- |
| BoHuV/DH/2022 | DH-1F  DH-IR | GGGACCACCACTTTCAGTGAC  GTCCAGGTCCTTCAAACTCAAAG | 872 bp |
|  | DH-2F  DH-2R | GCAGTCAATATGGAGATTCCA  TCCAGTCTCTGCAGGTGTAATC | 1786 bp |
|  | DH-3F  DH-3R | AGTTGGTAGTGGTGGCTATTTTAC  GTCTCCTGAATTCACTGAGAGT | 1389 bp |
|  | DH-4F  DH-4R | TATGGTGTGATTCTTTCCCGTGC  ACAACTGGTTGCTGTTGATATCC | 1038 bp |
|  | DH-5F  DH-5R | ATGGCAAGCATACCACATGAGA  AGAGCTCCCGTCTCAAACTCG | 1180 bp |
|  | DH-6F  DH-6R | TACAGTGGMAYCGCTGTTGTG  CATCATGAGCATCAGTTCGGC | 1139bp |
|  | DH-7F  DH-7R | AATGAACGACTGTCGCTGCACG  TTCAAGRTCATCAAAGCRGGGAAC | 1079 bp |
|  | DH-8F  DH-8R | TCGTACCAGAAGTGTATGATGGC  TTACTCTGGGGAAAATTAACCCT | 550 bp |

Supplementary Table 2 Amplification primers for the whole genome of five Hunnivirus strains（Continued）

| Virus name | Primer | Primer sequence ( 5 ' -3 ' ) | Product size |
| --- | --- | --- | --- |
| BoHuV/YZ/2022 | YZ-1F  YZ-IR | GGGACCACCACTTTCAGTGAC  AAAAGTCTTCTGGCGTCTGACCA | 851 bp |
|  | YZ-2F  YZ-2R | GGTACAGCTTCATTGTTGCTAGA  GCCATGGAATTCAGCTTCAAGTGG | 1158 bp |
|  | YZ-3F  YZ-3R | GTGGCTGGCATGTGCAAGTGCAA  CTACATTCAGTACATCTGCAAGG | 1241 bp |
|  | YZ-4F  YZ-4R | GGTGGGTATTTTACAATTTGGCA  GCAAATCTTGGCACACAGCCGA | 1184 bp |
|  | YZ-5F  YZ-5R | TATGGTGTGATTCTTTCTCGTGC  TTCATGTGGTATGCTTGCCATA | 872 bp |
|  | YZ-6F  YZ-6R | TGGTTTGATGAGATGCGGAAA  GGCTTCAATGGATCTGGTCCTG | 975 bp |
|  | YZ-7F  YZ-7R | TGGTGTGACCTTGCAATYGCAC  GTATGACTGCCCTGCTTCAACT | 1507 bp |
|  | YZ-8F  YZ-8R | AATGAACGACTGTCGCTGCACG  TTCAAGRTCATCAAAGCRGGGAAC | 1079 bp |
|  | YZ-9F  YZ-9R | CACTCGTACCAGAAGTGTATG  TTACTCTGGGGAAAATTAACCCT | 551 bp |
